# Supplementary material for: Modeling of three-dimensional innervated epidermal like-layer in a microfluidic chip-based coculture system
Source: Nat Commun. 2023 Mar 17;14:1488. doi: 10.1038/s41467-023-37187-4 (PMC10023681; doi:10.1038/s41467-023-37187-4)
Supplement: Supplementary file 1 — Supplementary information [file 41467_2023_37187_MOESM1_ESM.pdf]

Supplementary Figures

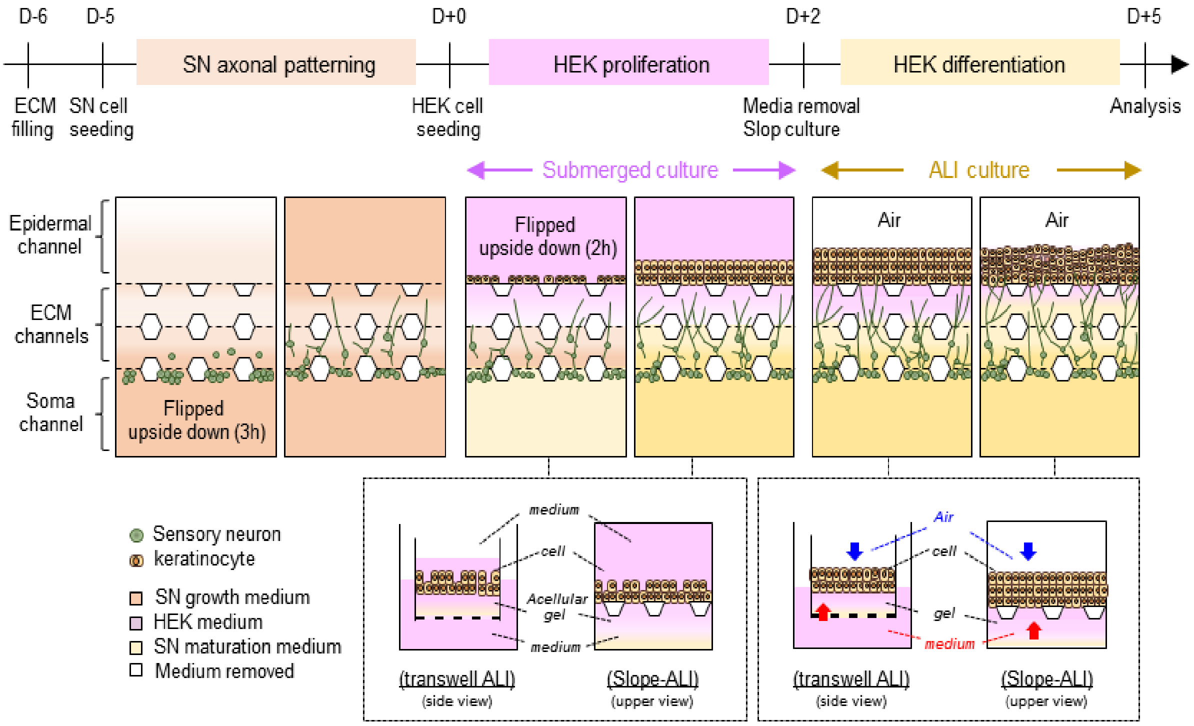

Supplementary Figure 1. Microfluidic platform and culture system for sensory neurons-keratinocytes co-culture. Experimental workflow of cell seeding and culture for generating the innervated epidermal chip (upper). Schematic and direct comparison of the air-liquid interface (ALI) culture protocol between conventional transwell systems and our microfluidic chip systems (lower).

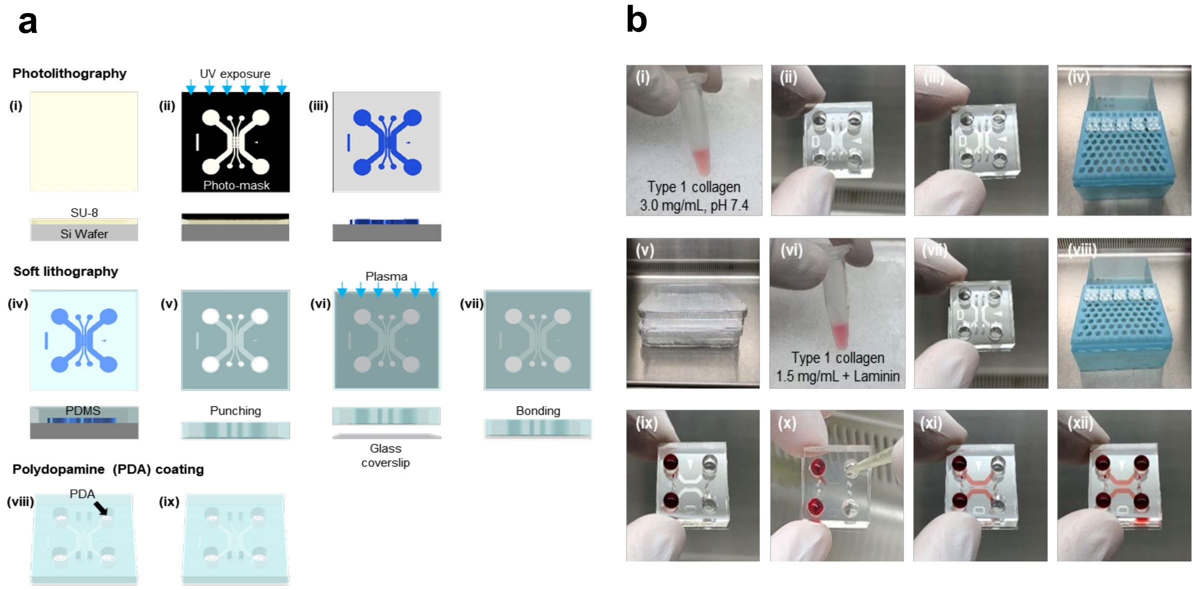

Supplementary Figure 2. Schematic diagram of the fabrication and gel-filling process of the microfluidic chip. a. Fabrication process. A microfluidic device incorporating hydrogel was fabricated from PDMS (Sylgard 184; Dow Chemical, Midland, MI, USA) cured on a SU-8 patterned silicon wafer by conventional soft lithography. Cured PDMS was cut, and holes were constructed with a biopsy punch to form the device, which was autoclaved at 120 °C for 20 min followed by overnight drying at 80 °C. The PDMS device and a cover glass were bonded together by oxygen plasma treatment (Femto Science, Yongin, Korea). The microchannels were coated with 2 mg/ml polydopamine (PDA) solution for 2 h at 25 °C, washed with distilled deionized water, and dried overnight at 80 °C to render the channel surface hydrophobic. The dimensions of the microfluidic device are illustrated in Fig. 1a. **b.** Gel-filling procedure. PDA-coated microfluidic devices were prepared before the gel-filling procedure. Type I collagen solution (3.0 mg/ml, pH 7.4) was prepared on ice. The keratinocyte channel side gel channel was filled with the type I collagen solution and placed in the pre-warmed humid chamber containing sterilized DI water and incubated under 5% CO2 at 37 °C for 30 min for gelation. For the subsequent gel filling step, a pre-prepared mixture of collagen (1.5 mg/ml, pH 7.4) and laminin was filled in the sensory neuron-side gel channel and gelled in the same manner. Each cell channel was filled with media of each cell.

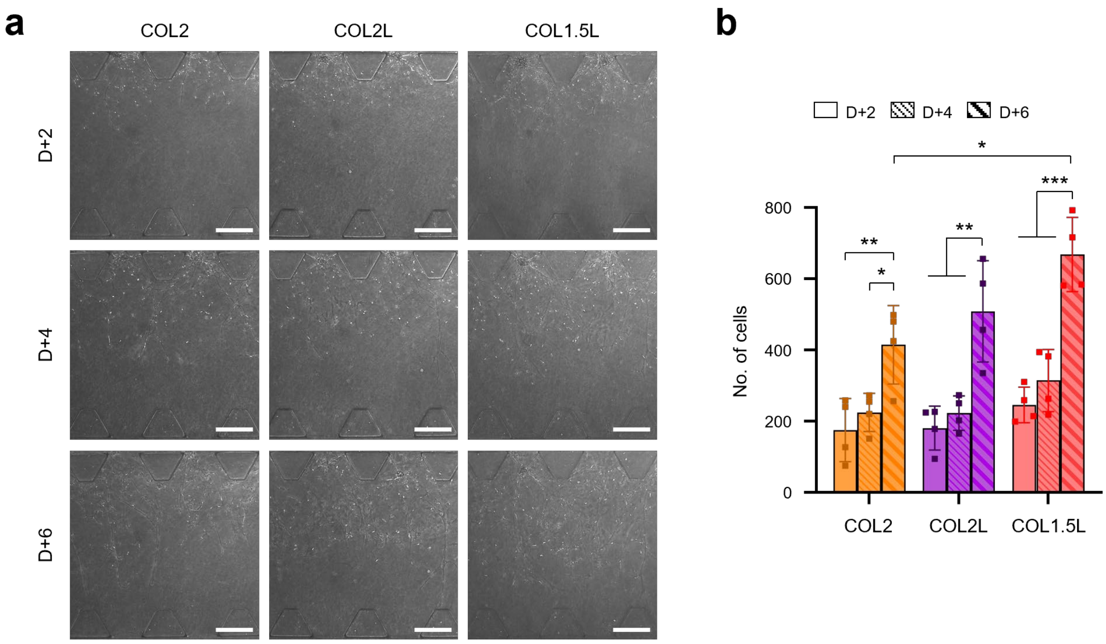

Supplementary Figure 3. The effect of ECM conditions on sensory neurons on the microfluidic chip. a. Time-lapse phase-contrast images of sensory neurons cultured in the COL2, COL2L, or COL1.5L gel conditions for 6 days. COL 2; collagen I at 2mg/ml concentration, COL 2L; collagen I at 2mg/mL with 10% laminin, COL 1.5L; collagen I at 1.5mg/mL with 10% laminin. Scale bars; 100 µm. **b.** The number of cells that migrated into the collagen gel (n = 4 devices, D+6 vs D+2 **p=0.0093, D+6 vs D+4 *p=0.0317 for COL2, D+6 vs D+2 **p=0.002, D+6 vs D+4 **p=0.005 for COL2L, D+6 vs D+2 ***p=0.0001, D+6 vs D+4 ***p=0.0005 for COL1.5L, COL1.5L(D+6) vs COL2(D+6) *p=0.0367, 2 independent replicates). Data are mean ± s.d. One-way ANOVA, Tukey's multiple comparisons test. *$p < 0.05$, **$p < 0.01$, ***$p < 0.001$.

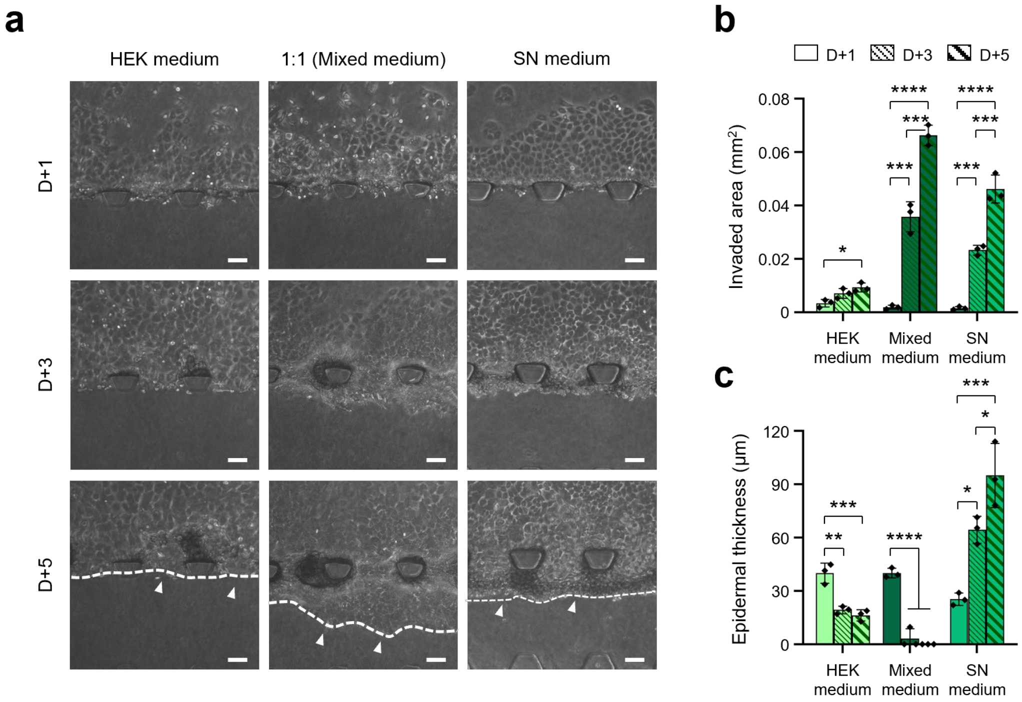

Supplementary Figure 4. Optimization of keratinocyte culture medium in the microfluidic chip. a. Time-lapse phase-contrast images of sensory neurons cultured for 5 days in the keratinocyte medium, sensory neuron medium, or each 1:1 mixed medium. The dashed lines indicate cells that invaded the ECM gel layer and the arrowheads indicate the boundary between the keratinocyte layer and the ECM layer. Scale bars; 100 μm. **b.** Quantification of the invaded keratinocytes (n = 3 devices, D+5 vs D+1 *p=0.0109 for HEK medium, D+5 vs D+1 ****p<0.0001, D+5 vs D+3 ***p=0.0002, D+3 vs D+1 ***p=0.0001 for Mixed medium, D+5 vs D+1 ****p<0.0001, D+5 vs D+3 ***p=0.0003, D+3 vs D+1 ***p=0.0004 for SN medium, 2 independent replicates) **c.** The epidermal thickness after culture in different medium conditions (n = 3 devices, D+5 vs D+1 ***p=0.0008, D+3 vs D+1 **p=0.0016 for HEK medium, D+5 vs D+1 ****p<0.0001, D+3 vs D+1 ****p<0.0001 for Mixed medium, D+5 vs D+1 ***p=0.0007, D+5 vs D+3 *p=0.0393, D+3 vs D+1 *p=0.0138 for SN medium, 2 independent replicates). Data are mean ± s.d. One-way ANOVA, Tukey's multiple comparisons test. *$p < 0.05$, **$p < 0.01$, ***$p < 0.001$, ****$p < 0.0001$.

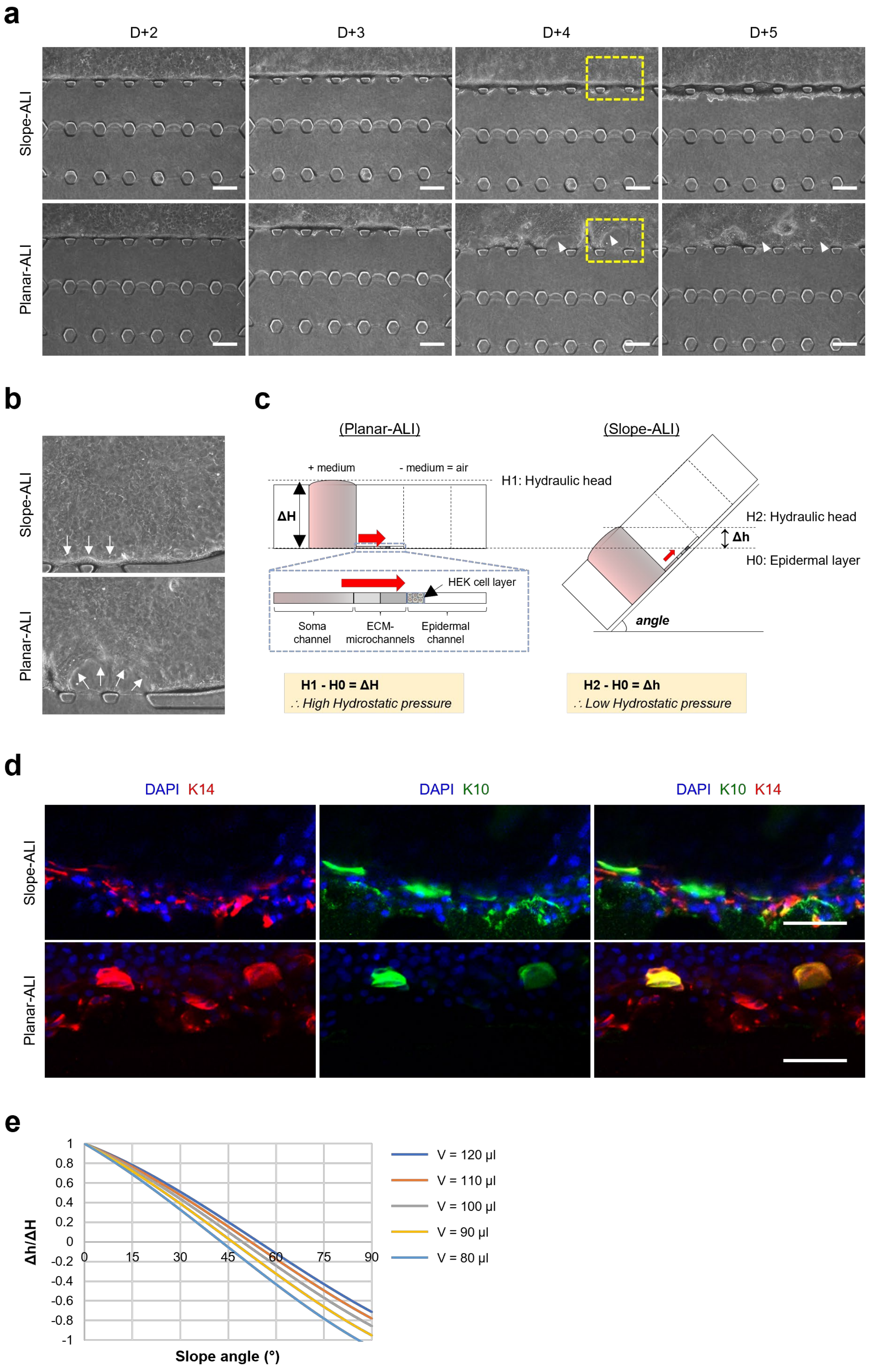

Supplementary Figure 5. The effect of the slope on epidermal development in the air-liquid interface (ALI) microfluidic chip. a. Time-lapse phase-contrast images of the epidermal development according to the slope of

the ALI chip. In the tilted condition of the ALI chip, the epidermal layer slightly invaded the ECM gel layer and was linearized near the interface between the keratinocyte and the ECM layer. Whereas, it is pushed toward the keratinocyte layer in the planar condition of the ALI chip. Scale bars; 250 μm. **b.** Magnifications of the region highlighted by the yellow dashed box in (**a**). Arrows indicate the epidermal layers in the ALI chip. **c**. Hypothetical illustration of hydrostatic pressure in ALI microfluidic chip system according to the slope. The two-ways black arrows represent the difference of hydraulic heads, and the red arrows represent the direction and magnitude of net hydrostatic pressure. **d.** Representative fluorescence images of K14 (red) and K10 (green) expression in epidermal layers of each group (2 independent replicates). Scale bars; 100 μm. **e**. Plots of hydraulic head difference ratio between slope and planar culture. Volume(V) represents the media volume in the SN soma channel and reservoirs.

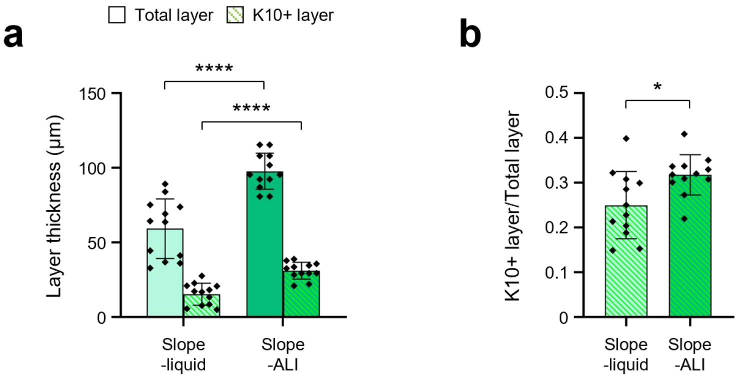

Supplementary Figure 6. The effect of the air-liquid interface (ALI) on epidermal development in the slope-based culture system. a. The epidermal thickness according to the presence (slope-liquid) or absence (slope-ALI) of the medium in the keratinocyte channel*n* = 12 ROIs, 3 ROIs per device, Slope-liquid vs Slope-ALI ****p<0.0001 for total layer and K10+ layer thickness, 2 independent replicates. **b.** The thickness of the differentiated epidermal layer ($K10^{+}$) relative to the total epidermal layer. *n* = 12 ROIs, 3 ROIs per device, *p=0.0137 for K10+ layer ratio, *2* independent replicates. Data are mean ± s.d., *$p < 0.05$, ****$p < 0.0001$. One-way ANOVA, Tukey's multiple comparisons test.

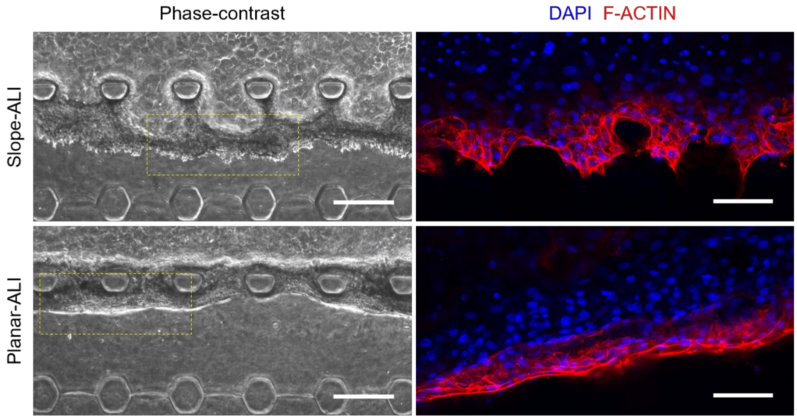

Supplementary Figure 7. The bifurcated rete ridges (RR) - like structures of the epidermal layer cultured in the slope-ALI microfluidic chip system. Representative phase-contrast images (left) and fluorescence images (right) of epidermal layers stained with F-ACTIN (red) and DAPI (blue). Right panels are magnifications of the region highlighted by the yellow dashed box in the left panels (2 independent replicates). Scale bars; 250 μm and 100 μm, respectively.

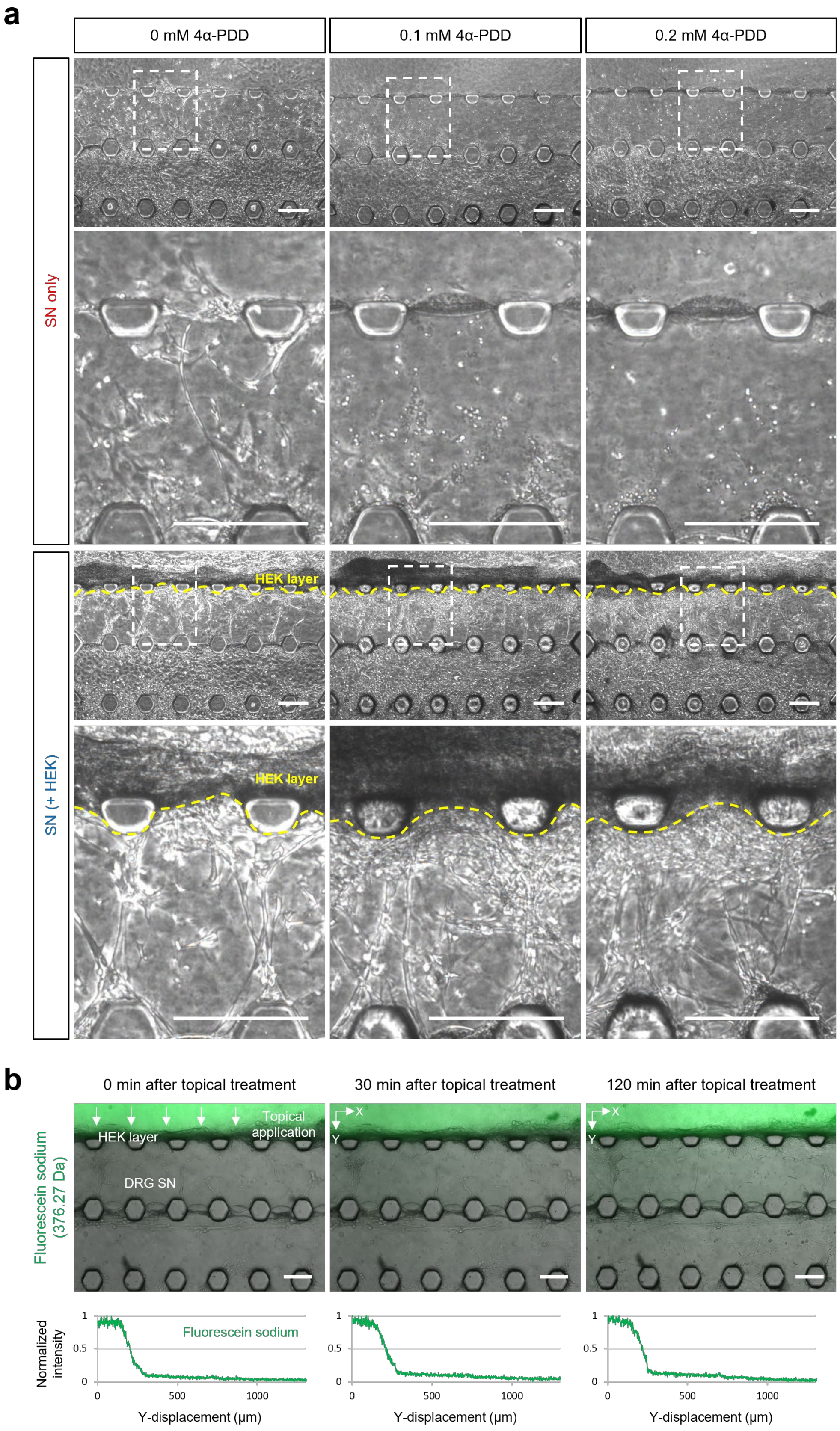
a
0 mM 4α-PDD
0.1 mM 4α-PDD
0.2 mM 4α-PDD
SN only
SN (+ HEK)
HEK layer
b
0 min after topical treatment
30 min after topical treatment
120 min after topical treatment
Fluorescein sodium (376.27 Da)
Topical application
HEK layer
DRG SN
X
Y
Normalized intensity
Fluorescein sodium
Y-displacement (μm)
0
0.5
1
500
1000

Supplementary Figure 8. The protective effect of the epidermal layer on sensory neurons of the innervated epidermal chip. Morphological images of sensory neurons after topical application of 4α-PDD (0.1 mM or 0.2 mM) to keratinocyte channel in innervated epidermal chips. Sensory neurons cultured alone (SN only) were damaged 2 h after 4α-PDD treatment, but sensory neurons of innervated epidermal chips (SN+HEK) were healthy and protected by the epidermal barrier. Magnified images of white dashed boxes in each upper panel. Yellow dashed lines indicate the boundary between keratinocytes and ECM layer (3 independent replicates). Scale bars; 250 µm. **b.** Fluorescence images of innervated epidermis at D+5 after topical treatment of fluorescein sodium (MW: 376.27 Da) (top) and normalized intensity of fluorescein sodium. Scale bars; 200 µm.

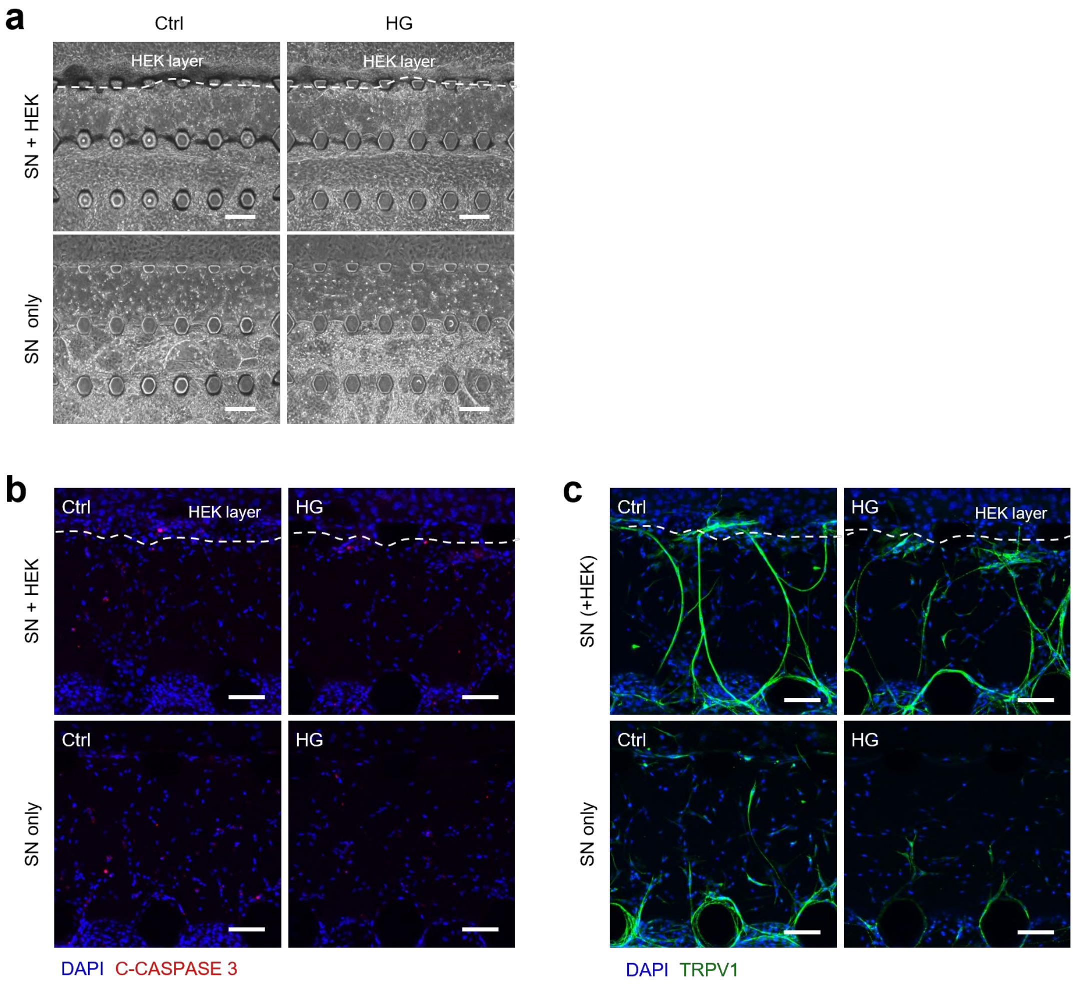

Supplementary Figure 9. Hyperglycemic effects on innervated epidermal chips. a. Representative phase-contrast images of sensory neurons alone (SN only) and innervated epidermis (SN + HEK) cultured for 3 days under control or high glucose condition (25 mM or 100 mM, respectively). Dashed lines indicate the boundary between keratinocytes and ECM layer. Scale bars; 250 µm. **b.** Fluorescence images of apoptotic cell assays of each group stained with the cleaved caspase 3 antibody (C-CASPASE 3, red) and DAPI (blue). Scale bars; 100 µm. **c.** Fluorescence images of TRPV1^{+} sensory neurons (green) of each group. DAPI (blue). Dashed lines indicate the boundary between keratinocytes and ECM layer (2 independent replicates). Scale bars; 100 µm.

Supplementary Table 1. 2D or 3D in vitro models for co-culture of sensory neurons and keratinocytes.

In vitro models of Sensory-Skin interaction			2D												this work
Reference number			[1]	[2]	[3]	[4]	[5]	[6]	[7]	[8]	[9]	[10]	[11]	[17]	
Year			2004	2007	2009	2009	2010	2011	2012	2013	2012	2013	2014	2016	2022
Cell type	Peripheral sensory neurons	DRG	M	R	R	M	P	R		P	P	R, M	R	P	R
	Epidermal cells	Keratinocyte	H	R	R	M	P	H	H	P	H	R	H	H	H
	Dermal cells	Fibroblast									H				
		Endothelial cell													
	Other cells			A431, ND7-23	A431, ND7-23	HEK293			F-11		Atopic keratinocyte				
Phenotypic analysis	Pan-neuronal markers	PGP 9.5													
		Neurofilament													
		SMI 312													
		Peripherin													
		β-III tubulin (Tuj1)													
	Peripheral sensory neuron subtypes	NF200													
		TrkB													
		TRPV1													
		CGRP / SP													
		TrkA													
		IB4													
	Histology	Masson's trichrome													
		Hematoxylin & Eosin													
	Basal layer properties (epidermis)	Ki-67													
		K5													
		K14													
	Differentiated layer properties (epidermis)	K1													
		K10													
		Involucrin													
		Loricrin													
		Filaggrin													
	Receptor expression (epidermis)	TRPV1													
		TRPV4													
Functional analysis	ELISA	CGRP concentration							Basal Cap						Basal Cap 4α-PDD
		SP concentration					Basal Cap		Basal Cap						Basal
	Calcium imaging ATP imaging (A)		Mech Mech (A) ATP UTP	ATP Cap		ATP Heat (A)		Mech		Mech Cap		Cap			Cap
	Electrophysiology					Heat ATP						Elec			
	Permeability test (Barrier function)														

2D substrate   Teflon divider   Microfluidic device

In vitro models of Sensory-Skin interaction			3D													this work
Reference number			[6]	[12]	[13]	[14]	[15]	[16]	[17]	[18]	[19]	[20]	[21]	[22]	[23]	
Year				2003	2009	2013	2014	2015	2016	2016	2017	2018	2019	2019	2022	2022
Cell type	Peripheral sensory neurons	DRG	R	M	M	P	M	M	P	P	R	H, M				R
	Epidermal cells	Keratinocyte	H	H	H	H	H	H	H	H	H	H	H	H	H	H
	Dermal cells	Fibroblast		H	H	H	H	H	H	H	H	H	H	H		
		Endothelial cell		H	H		H	H				H				
	Other cells				Schwann cell	Atopic keratinocyte				Diabetic fibroblast, keratinocyte		Schwann cell	hiNSC	hiNSC	hNSC	
Phenotypic analysis	Pan-neuronal markers	PGP 9.5														
		Neurofilament														
		SMI 312														
		β-III tubulin (Tuj1)														
	Peripheral sensory neuron subtypes	NF200														
		TRPV1														
		CGRP / SP														
		IB4														
	Histology	Masson's trichrome														
		Hematoxylin & Eosin														
	Basal layer properties (epidermis)	Ki-67														
		K5														
		K14														
	Differentiatied layer properties (epidermis)	K1														
		K10														
		Involucrin														
		Loricrin														
		Filaggrin														
	Receptor expression (epidermis)	TRPV1														
		TRPV4														
Functional analysis	ELISA	CGRP concentration				Basal			Basal Cap			Basal Cap				Basal Cap 4α-PDD
		SP concentration				Basal	Basal					Basal Cap				Basal
	Calcium imaging		Mech						Menthol MPD		Cap					Cap
	Permeability test (Barrier function)															

3D aggregate   3D skin equivalent   Microfluidic device

H; human, P; porcine, R; rat, M; mouse, hiNSC; human induced neural stem cell, Basal; unstimulated condition (basal level), Cap; capsaicin, 4α-PDD; 4α-phorbol 12,13-didecanoate, ATP; adenosine triphosphate, UTP; uridine triphosphate, Mech; mechanical stimulation, Elec; electrical stimulation, Heat; Heat stimulation. Colors; Yellow: 2D substrates culture model, Gray: Teflon divider culture model, Red: 3D aggregate model, Green: 3D skin equivalent model, Blue: Microfluidic chip culture model

Supplementary Table 2. List of antibodies and primers used in this study.

Antibody	Dilution	Company, Catalogue number
Mouse anti-neurofilament medium	1:500	Abcam, ab7794
Rabbit anti-PGP9.5	1:500	Abcam, ab108986
Rabbit anti-Tuj1	1:200	Sigma, T2200
Rabbit anti-NF200	1:100	Sigma, N4142
Mouse anti-TRPV1	1:500	Abcam, ab203103
Sheep anti-CGRP	1:500	Abcam, ab22560
Mouse anti-CGRP	1:100	Abcam, ab81887
FITC-conjugated anti-IB4	1:200	Sigma, L2895
Rabbit anti-Gap-43	1:250	Sigma, AB5220
Rabbit anti-cytokeratin 10	1:150	Abcam, ab76318
Mouse anti-cytokeratin 14	1:200	Abcam, ab7800
Mouse anti-cytokeratin 5	1:50	Santa Cruz, sc-32721
Rabbit anti-Ki67	1:500	Abcam, ab15580
Rabbit anti-loricrin	1:500	Novus, NBP1-33610
Rabbit anti-TRPV1	1:1000	Abcam, ab3487
Rabbit anti-TRPV4	1:250	Abcam, ab191580
Rabbit anti-Cleaved Caspase-3	1:400	Cell Signaling, #9664
Goat anti-Mouse Alexa 488	1:500	Invitrogen, A11001
Goat anti-Rabbit Alexa 488	1:500	Invitrogen, A-11034
Donkey anti-Sheep Alexa 488	1:500	Invitrogen, A11015
Goat anti-Mouse Alexa 568	1:500	Invitrogen, A-11004
Goat anti-Rabbit Alexa 568	1:500	Invitrogen, A-11011

Gene	Forward primer	Reverse primer
KRT14	CCAGTTCTCCTCTGGATCGCAG	GATCTTCCAGTGGGATCTGTGTCCA
KRT10	AATGACCGCCTGGCTTCCTA	CCTGATGTGAGTTGCCATGCT
LORICRIN	TGCCAGCATCTTCTCTCCTCAC	CAGAGGTCTTCACGCAGTCCA
HPRT	CATTATGCTGAGGATTTGGAAAGG	CTTGAGCACACAGAGGGCTACA

Supplementary References

1. Koizumi, S., Fujishita, K., Inoue, K., Shigemoto-Mogami, Y., Tsuda, M. & Inoue, K. Ca2+ waves in keratinocytes are transmitted to sensory neurons: the involvement of extracellular ATP and P2Y2 receptor activation. *Biochem. J.* **380**, 329-338 (2004).

2. Ulmann L., Rodeau J. L., Danoux L., Contet-Audonneau J. L., Pauly G., Schlichter R. Trophic effects of keratinocytes on the axonal development of sensory neurons in a coculture model. *Eur J Neurosci.* **26**,113-125 (2007).

3. Ulmann L., Rodeau J. L., Danoux L., Contet-Audonneau J. L., Pauly G., Schlichter R. Dehydroepiandrosterone and neurotrophins favor axonal growth in a sensory neuron-keratinocyte coculture model. *Neuroscience* **159**, 514-525 (2009).

4. Mandadi, S., Sokabe, T., Shibasaki, K., Katanosaka, K., Mizuno, A., Moqrich, A., Patapoutian, A., Fukumi-Tominaga, T., Mizumura, K., Tominaga, M. TRPV3 in keratinocytes transmits temperature information to sensory neurons via ATP. *Pflugers Arch.* **458**, 1093-1102 (2009).

5. Pereira U., Boulais N., Lebonvallet N., Lefeuvre L., Gougerot A., Misery L. Development of an in vitro coculture of primary sensitive pig neurons and keratinocytes for the study of cutaneous neurogenic inflammation. *Exp Dermatol*. **19**, 931-935 (2010).

6. Tsutsumi M., Goto M., Denda S., Denda M. Morphological and functional differences in coculture system of keratinocytes and dorsal-root-ganglion-derived cells depending on time of seeding. *Exp Dermatol.* **20**, 464-467 (2011).

7. Le Gall-Ianotto C., Andres E., Hurtado S. P., Pereira U., Misery L. Characterization of the first coculture between human primary keratinocytes and the dorsal root ganglion-derived neuronal cell line F-11. *Neuroscience* **210**, 47-57 (2012).

8. Klusch A., Ponce L., Gorzelanny C., Schäfer I., Schneider S. W., Ringkamp M., Holloschi A., Schmelz M., Hafner M., Petersen M.. Coculture model of sensory neurites and keratinocytes to investigate functional interaction: chemical stimulation and atomic force microscope-transmitted mechanical stimulation combined with live-cell imaging. *J Invest Dermatol*. **133**, 1387-1390 (2013).

9. Roggenkamp D, Falkner S, Stäb F, Petersen M, Schmelz M, Neufang G. Atopic keratinocytes induce increased neurite outgrowth in a coculture model of porcine dorsal root ganglia neurons and human skin cells. *J Invest Dermatol.* **132**, 1892-1900 (2012).

10. Tsantoulas C., Farmer C., Machado P., Baba K., McMahon S. B., Raouf R. Probing functional properties of nociceptive axons using a microfluidic culture system. *PLoS One*. **8**, e80722 (2013).

11. Jun-Ichi K., Masashi N., Moe T., Makiko G., Sumiko D., Kentaro T., Mitsuhiro D. Coculture system of

keratinocytes and dorsal-root-ganglion-derived cells for screening neurotrophic factors involved in guidance of neuronal axon growth in the skin. *Exp Dermatol.* **23**, 58-60 (2014).

12. Gingras M., Bergeron J., Déry J., Durham H. D., Berthod F. In vitro development of a tissue-engineered model of peripheral nerve regeneration to study neurite growth. *FASEB J.* **17**, 2124-2126 (2003).

13. Mathieu B., Myriam G., François B. Improvement of nerve regeneration in tissue-engineered skin enriched with schwann cells. *J Invest Dermatol.* 129, 2895-900 (2009).

14. Roggenkamp D., Köpnick S., Stäb F., Wenck H., Schmelz M., Neufang G. Epidermal nerve fibers modulate keratinocyte growth via neuropeptide signaling in an innervated skin model. *J Invest Dermatol*. **133**, 1620-1628 (2013).

15. Mathieu B., Lorène M., Marie-Anne G., Sabrina B., Sébastien C., François B. Sensory neurons accelerate skin reepithelialization via substance P in an innervated tissue-engineered wound healing model. *Tissue Eng Part A.* **20**, 2180-8 (2014).

16. Sébastien C., Sabrina LO., Sabine P., Nicolas B., Valérie AF., François B. In vitro glycation of an endothelialized and innervated tissue-engineered skin to screen anti-AGE molecules. *Biomaterials.* **51**, 216-225 (2015).

17. Dennis R., Anne-Christin W., Melanie S., Horst W., Franz S., Gitta N. Menthoxypropanediol inhibits nerve growth factor-induced nerve fibre sprouting in coculture models of sensory neurons and skin cells. *Exp Dermatol.* **25**, 824-6 (2016).

18. Olga R., Thomas F., Gitta N., Martin S., Harald G., Volkhard K., Horst W., Franz S., Lara T., Ludger K., Dennis R. Impaired glyoxalase activity is associated with reduced expression of neurotrophic factors and pro-inflammatory processes in diabetic skin cells. *Exp Dermatol.* **26**, 44-50 (2017).

19. Martorina F., Casale C., Urciuolo F., Netti P. A., Imparato G. In vitro activation of the neuro-transduction mechanism in sensitive organotypic human skin model. *Biomaterials* **113**, 217-229 (2017).

20. Muller Q, Beaudet MJ, De Serres-Berard T, Bellenfant S, Flacher V, Berthod F. Development of an innervated tissue-engineered skin with human sensory neurons and Schwann cells differentiated from iPS cells. *Acta Biomater.* **82**, 93-101 (2018).

21. Sarah EVY., Kasey AT., Hanh N., Dana MC., David LK. Human Skin Equivalents Demonstrate Need for Neuro-Immuno-Cutaneous System. *Adv Biosyst.* **3**, e1800283 (2019).

22. Sarah ELV., Kasey AT., Hanh N., Rosalyn DA., Dana MC., David LK. 3D biomaterial matrix to support long term, full thickness, immuno-competent human skin equivalents with nervous system components. *Biomaterials.* **198**, 194-03 (2019).

23. Jong Sung L., Jin K., Baofang Cui 1, Su Kyeom K., Sun-A C., Susun A., Seung-Woo C. Hybrid skin chips for toxicological evaluation of chemical drugs and cosmetic compounds. *Lab Chip.* **22**, 343-353 (2022).

24. Yoojin S., Se Hoon C., Eunhee K., Enjana B., Jeong Ah K., Seok C., Doo Yeon K., Roger DK., Rudolph ET. Blood–Brain Barrier Dysfunction in a 3D In Vitro Model of Alzheimer's Disease. *Adv. Sci*. **6**, 1900962 (2019).

25. Youngkyu C., Kyuhwan N., Yesl J., Jihee W., Ji Hun Y., Seok C. Three-Dimensional In Vitro Lymphangiogenesis Model in Tumor Microenvironment. *Front. Bioeng. Biotechnol*. **9**, 697657 (2021).

26. Franziska K., Maximiliane W., Luisa K., Tobias M., Phillip F., Florian GB., Nurcan Ü. Patient-derived in vitro skin models for investigation of small fiber pathology. *Annals of Clin and Trans Neurol.* **6**, 1797-1806 (2019).

27. Alexandre Guichard, Noëlle Remoué, and Thibault Honegger. In Vitro Sensitive Skin Models: Review of the Standard Methods and Introduction to a New Disruptive Technology. *Cosmetics.* **9**, 67 (2022).
