# Supplementary material for: Modeling of three-dimensional innervated epidermal like-layer in a microfluidic chip-based coculture system
Source: Nat Commun. 2023 Mar 17;14:1488. doi: 10.1038/s41467-023-37187-4 (PMC10023681; doi:10.1038/s41467-023-37187-4)
Supplement: Supplementary file 2 — Reporting Summary [file 41467_2023_37187_MOESM2_ESM.pdf]

nature portfolio

Corresponding author(s): Seok Chung

Last updated by author(s): Feb 16, 2023

Reporting Summary

Nature Portfolio wishes to improve the reproducibility of the work that we publish. This form provides structure for consistency and transparency in reporting. For further information on Nature Portfolio policies, see our Editorial Policies and the Editorial Policy Checklist.

Statistics

For all statistical analyses, confirm that the following items are present in the figure legend, table legend, main text, or Methods section.

n/a	Confirmed	
☐	☒	The exact sample size (*n*) for each experimental group/condition, given as a discrete number and unit of measurement
☐	☒	A statement on whether measurements were taken from distinct samples or whether the same sample was measured repeatedly
☐	☒	The statistical test(s) used AND whether they are one- or two-sided<br>*Only common tests should be described solely by name; describe more complex techniques in the Methods section.*
☒	☐	A description of all covariates tested
☐	☒	A description of any assumptions or corrections, such as tests of normality and adjustment for multiple comparisons
☐	☒	A full description of the statistical parameters including central tendency (e.g. means) or other basic estimates (e.g. regression coefficient) AND variation (e.g. standard deviation) or associated estimates of uncertainty (e.g. confidence intervals)
☐	☒	For null hypothesis testing, the test statistic (e.g. *F*, *t*, *r*) with confidence intervals, effect sizes, degrees of freedom and *P* value noted<br>*Give P values as exact values whenever suitable.*
☒	☐	For Bayesian analysis, information on the choice of priors and Markov chain Monte Carlo settings
☒	☐	For hierarchical and complex designs, identification of the appropriate level for tests and full reporting of outcomes
☒	☐	Estimates of effect sizes (e.g. Cohen's *d*, Pearson's *r*), indicating how they were calculated

Our web collection on statistics for biologists contains articles on many of the points above.

Software and code

Policy information about availability of computer code

Data collection	Fluorescence imaging was performed using a confocal laser-scanning microscope (Olympus, Japan) (LSM 700, Carl Zeiss, Germany) fluorescence microscope (Carl Zeiss, Germany), Fluoview (Olympus, Japan) and ZEN 2.3 software (Carl Zeiss, Germany).<br>The absorption of rat CGRP Enzyme Immunoassay kit was measured using VICTOR X3 (PerkinElmer, USA) at a wavelength of 405 nm.<br>qRT-PCR was performed using Power SYBR Green PCR Master Mix (Applied Biosystems) in the StepOne Real-time PCR equipment (Applied Biosystems).
Data analysis	The obtained fluorescence images were analyzed using the Image J software (https://imagej. nih.gov/ij/inde x.html), ZEN 2.3 software and Microsoft 365 PowerPoint (Microsoft, USA).<br>Quantification and the statistical calculations of the results were carried out by Microsoft 365 Excel (Microsoft, USA) and Prism software (GraphPad Software, San Jose, CA, USA).

For manuscripts utilizing custom algorithms or software that are central to the research but not yet described in published literature, software must be made available to editors and reviewers. We strongly encourage code deposition in a community repository (e.g. GitHub). See the Nature Portfolio guidelines for submitting code & software for further information.

Data

Policy information about availability of data

All manuscripts must include a data availability statement. This statement should provide the following information, where applicable:

- Accession codes, unique identifiers, or web links for publicly available datasets
- A description of any restrictions on data availability
- For clinical datasets or third party data, please ensure that the statement adheres to our policy

All data needed to evaluate the conclusions in the article are presented in the manuscript and/or the Supplementary information. Raw data for all figures are provided in Source Data file. Additional data related to this paper is available from the corresponding author upon reasonable request.

Human research participants

Policy information about studies involving human research participants and Sex and Gender in Research.

Reporting on sex and gender	N/A
Population characteristics	N/A
Recruitment	N/A
Ethics oversight	N/A

Note that full information on the approval of the study protocol must also be provided in the manuscript.

Field-specific reporting

Please select the one below that is the best fit for your research. If you are not sure, read the appropriate sections before making your selection.

[x] Life sciences [ ] Behavioural & social sciences [ ] Ecological, evolutionary & environmental sciences

For a reference copy of the document with all sections, see nature.com/documents/nr-reporting-summary-flat.pdf

Life sciences study design

All studies must disclose on these points even when the disclosure is negative.

Sample size	No statistical methods were used to predetermine sample sizes. Throughout the study, the sample size was determined based on our preliminary studies and on the criteria in the field. At least 3 biological replicates per group were included for each experiment, and 1-4 independent experiments were performed. Numbers of biological replicates and independent replicates were shown in the figure captions.
Data exclusions	No data were excluded.
Replication	The number of experiments performed is noted in each figure caption. Experiments were independently repeated at least two times. Similar results were observed in all repeated trials for each experiment.
Randomization	Randomization was not applicable in this study. All samples were prepared under the same conditions in each experiment.
Blinding	The samples for this study were prepared under the same conditions. Quantification of obtained images, qRT-PCR, ELISA were performed in blind.

Reporting for specific materials, systems and methods

We require information from authors about some types of materials, experimental systems and methods used in many studies. Here, indicate whether each material, system or method listed is relevant to your study. If you are not sure if a list item applies to your research, read the appropriate section before selecting a response.

Materials & experimental systems

n/a	Involved in the study	
	x	Antibodies
	x	Eukaryotic cell lines
x		Palaeontology and archaeology
	x	Animals and other organisms
x		Clinical data
x		Dual use research of concern

Methods

n/a	Involved in the study	
x		ChIP-seq
x		Flow cytometry
x		MRI-based neuroimaging

Antibodies

Antibodies used

[Primary antibodies]
Mouse anti-neurofilament medium (1:500 dilution; Abcam, ab7794)
Rabbit anti-PGP9.5 (1:500 dilution; Abcam, ab108986)
Rabbit anti-Tuj1 (1:200 dilution; Sigma, T2200)
Rabbit anti-NF200 (1:100 dilution; Sigma, N4142)
Mouse anti-TRPV1 (1:500 dilution; Abcam, ab203103)
Sheep anti-CGRP (1:500 dilution; Abcam, ab22560)
Mouse anti-CGRP (1:100 dilution; Abcam, ab81887)
FITC-conjugated anti-IB4 (1:200 dilution; Sigma, L2895)
Rabbit anti-Gap-43 (1:250 dilution; Sigma, AB5220)
Rabbit anti-cytokeratin 10 (1:150 dilution; Abcam, ab76318)
Mouse anti-cytokeratin 14 (1:200 dilution; Abcam, ab7800)
Mouse anti-cytokeratin 5 (1:50 dilution; Santa Cruz, sc-32721)
Rabbit anti-Ki67 (1:500 dilution; Abcam, ab15580)
Rabbit anti-loricrin (1:500 dilution; Novus, NBP1-33610)
Rabbit anti-TRPV1 (1:1000 dilution; Abcam, ab3487)
Rabbit anti-TRPV4 (1:250 dilution; Abcam, ab191580)
Rabbit anti-Cleaved Caspase-3 (1:400 dilution; Cell Signaling, #9664)

[Secondary antibodies]
Goat anti-Mouse Alexa 488 (1:500; Invitrogen, A-11001)
Goat anti-Rabbit Alexa 488 (1:500; Invitrogen, A-11034)
Donkey anti-Sheep Alexa 488 (1:500, Invitrogen, A11015)
Goat anti-Mouse Alexa 568 (1:500; Invitrogen, A-11004)
Goat anti-Rabbit Alexa 568(1:500; Invitrogen, A-11011)

Validation

All antibodies listed above are commercially available and have been verified by many references provided on the website of the companies that sell antibodies.

Mouse anti-neurofilament medium (1:500 dilution; Abcam, ab7794) reacts with Mouse, Rat, Cow, Cat, Human, Pig and validated for use in ICC/IF, as stated on the Abcam website.
Rabbit anti-PGP9.5 (1:500 dilution; Abcam, ab108986) reacts with Mouse, Rat, Human and validated for use in ICC/IF, as stated on the Abcam website.
Rabbit anti-Tuj1 (1:200 dilution; Sigma, T2200) reacts with rat, human, mouse and validated for use in Immunofluorescence staining, as stated on the Sigma-Aldrich website.
Rabbit anti-NF200 (1:100 dilution; Sigma, N4142) reacts with wide range of species including rat, mouse and validated for use in Immunohistochemical staining, as stated on the Sigma-Aldrich website. Several published papers have used this antibody for immunostaining with rat and mouse DRG sensory neurons [Front Cell Neurosci. 2015; 9: 298., Neuron. 2019 Aug 21;103 (4):598-616.e7.].
Mouse anti-TRPV1 (1:500 dilution; Abcam, ab203103) reacts with Mouse, Rat and validated for use in ICC/IF, as stated on the Abcam website.
Sheep anti-CGRP (1:500 dilution; Abcam, ab22560) reacts with Rat, Human and validated for use in ICC/IF, as stated on the Abcam website.
Mouse anti-CGRP (1:100 dilution; Abcam, ab81887) reacts with Mouse, Rat, Guinea pig, Human and validated for use in ICC/IF, as stated on the Abcam website.
FITC-conjugated anti-IB4 (1:200 dilution; Sigma, L2895) reacts with rat, human, mouse DRG neurons and Several published papers have used this antibody for immunostaining with rat and mouse DRG sensory neurons [J Physiol Pharmacol. 2017 Jun;68(3):385-395., Cell Rep. 2019 Apr 2;27(1):71-85.e3., Pain. 2014 Mar;155(3):606-616.].
Rabbit anti-Gap-43 (1:250 dilution; Sigma, AB5220) reacts with wide range of species including mouse (high degree), rat, bovine, chicken, human and validated for use in Immunohistochemical staining, as stated on the Sigma-Aldrich website.
Rabbit anti-cytokeratin 10 (1:150 dilution; Abcam, ab76318) reacts with Mouse, Rat, Human and validated for use in ICC/IF, as stated on the Abcam website.
Mouse anti-cytokeratin 14 (1:200 dilution; Abcam, ab7800) reacts with Human and validated for use in ICC, as stated on the Abcam website.
Mouse anti-cytokeratin 5 (1:50 dilution; Santa Cruz, sc-32721) reacts with mouse, rat and human and validated for use in IF, as stated

on the Santa Cruz website.
Rabbit anti-Ki67 (1:500 dilution; Abcam, ab15580) reacts with Mouse, Human and validated for use in ICC/IF, as stated on the Abcam website.
Rabbit anti-loricrin (1:500 dilution; Novus, NBP1-33610) reacts with Human, Mouse, Rhesus Macaque and validated for use in ICC/IF, as stated on the Novus Biologicals website.
Rabbit anti-TRPV1 (1:1000 dilution; Abcam, ab3487) reacts with Human and validated for use in Immunohistochemical staining, as stated on the Abcam website.
Rabbit anti-TRPV4 (1:250 dilution; Abcam, ab191580) reacts with Human and validated for use in ICC/IF, as stated on the Abcam website.
Rabbit anti-Cleaved Caspase-3 (1:400 dilution; Cell Signaling, #9664) reacts with Human, Mouse, Rat, Monkey and validated for use in IF, as stated on the Cell Signaling Technology website.

Eukaryotic cell lines

Policy information about cell lines and Sex and Gender in Research

Cell line source(s)	Adult normal human epidermal keratinocytes (NHEK) (Lonza, 00192627)
Authentication	Sensory neurons from rat embryonic DRG were authenticated with immunostaining of neuronal markers (NF-M, Tuj1, PGP9.5) and sensory neuronal markers (TRPV1, CGRP, IB4, NF200). Human epidermal keratinocyte cell lines (NHEK) were authenticated with immunostaining of epidermal markers (K14, K10, Loricrin).
Mycoplasma contamination	It was confirmed that all animals were negative for mycoplasma infection. Mycoplasma testing was performed using e-Myco™ plus Mycoplasma PCR Detection Kit from iNtRON Biotechnology. Cell lines were negative for mycoplasma contamination.
Commonly misidentified lines (See ICLAC register)	No misidentified cell lines were used in this study.

Animals and other research organisms

Policy information about studies involving animals; ARRIVE guidelines recommended for reporting animal research, and Sex and Gender in Research

Laboratory animals	Dorsal root ganglion (DRG) were isolated from DRG of embryonic day 15 (E15) Sprague-Dawley rat embryos (KOATECH, Gyeonggi, South Korea).
Wild animals	No wild animals were used in this study.
Reporting on sex	Rat embryos (E15) of all sexes were used in this study.
Field-collected samples	No field-collected samples were used in this study.
Ethics oversight	All the animal procedures were approved by the Korea University Institutional Animal Care and Use Committee (KUIACUC-2017-138).

Note that full information on the approval of the study protocol must also be provided in the manuscript.
